# Radiologic finding of intraosseous gas: A rare case of emphysematous osteomyelitis of the foot

**DOI:** 10.1016/j.radcr.2022.11.007

**Published:** 2022-12-01

**Authors:** Vikash Bhattarai, Kshitiz Acharya, Sandip Kuikel, Sandeep Mahat, Surbhi Agarwal, Raman Ghimire, Astha Shree Krishna Poudel

**Affiliations:** a Department of Radiodiagnosis and Imaging, National Academy of Medical Sciences, Bir Hospital, Kathmandu, 44600, Nepal; b Maharajgunj Medical Campus, Tribhuvan University Institute of Medicine, Kathmandu, Nepal

**Keywords:** Emphysematous osteomyelitis, Intraosseous gas, Foot, Case report

## Abstract

Emphysematous osteomyelitis is a rare but potentially life-threatening infection which is characterized by presence of intraosseous gas. It is a very rare form of osteomyelitis which is complicated by infection with gas forming organisms. In majority of the cases, it has been found to be associated with comorbidities like immunosuppressive therapies, diabetes mellitus, alcohol use, and several others. Early identification on radiologic imaging is necessary to enable implementation of prompt treatment plan.

## Introduction

Emphysematous osteomyelitis is a rare condition, characterized by presence of intraosseous gas, identified on imaging, in absence of direct communication between bone and the external environment due to entities like compound fracture, or recent open surgery [1,2]. It was first described by Ram et al. in 1981 as a sign of osteomyelitis on computed tomography scan (CT scan) [2,3]. In majority of the cases, it is found to be associated with multiple comorbidities like diabetes mellitus, immunosuppressive therapy, malignancy, alcohol abuse, Crohn's disease, and several other conditions [1]. The majority of the reported cases have been found to involve the axial skeleton, whereas only few of the cases reported have been found to involve the appendicular skeleton, mainly femur, tibia, and the foot [1].

We herein, report a case of emphysematous osteomyelitis of the foot in a 59-year-old diabetic and hypertensive male patient.

## Case presentation

Our patient is a 59-year-old male who is a current smoker and alcohol consumer with a history of having comorbidities like type II diabetes mellitus and hypertension for 20 years, and currently not under medication. The patient presented to the emergency department of our center with the complaint of having a traumatic wound on his right great toe 6 months back, which never healed, rather gradually progressed to reach up to the ankle. The wound was associated with severe cramping type of continuous pain, with blackish discoloration of overlying skin for the last 10 days along with fowl smelling from the foot. The patient also stated that he had intermittent fever for the last 1 month with maximum recorded temperature at emergency being 101-degree Fahrenheit. Other vital parameters were normal in range at presentation with blood pressure: 110/70 mmHg, heart rate: 92 beats/min and respiratory rate: 14/min.

His blood investigation revealed leukocytosis with neutrophils predominance (90% neutrophils with total leukocyte count: 28,000/cumm, N: 4000-11,000). On biochemical examination, his Bilirubin (direct and total), SGOT and SGPT were within the normal range, but his alkaline phosphatase was found to be slightly elevated (161, N: 3-150). His serum LDH level was also found to be high (607, N: 225-450 IU/L). His HbA1c level was found to be 12.9% (N: 4.0-6.5%). Blood culture for was done, and *Escherichia coli* was isolated, and was sensitive to gentamycin and tigecycline and resistant to amoxycillin, amoxiclav, cefipime, ceftriaxone, cotrimoxazole, doxycycline, ofloxacin, and piperacillin/tazobactum. His urine routine and microscopic examination was found to be normal, and urine culture revealed no growth of organisms.

**Fig. 1:**
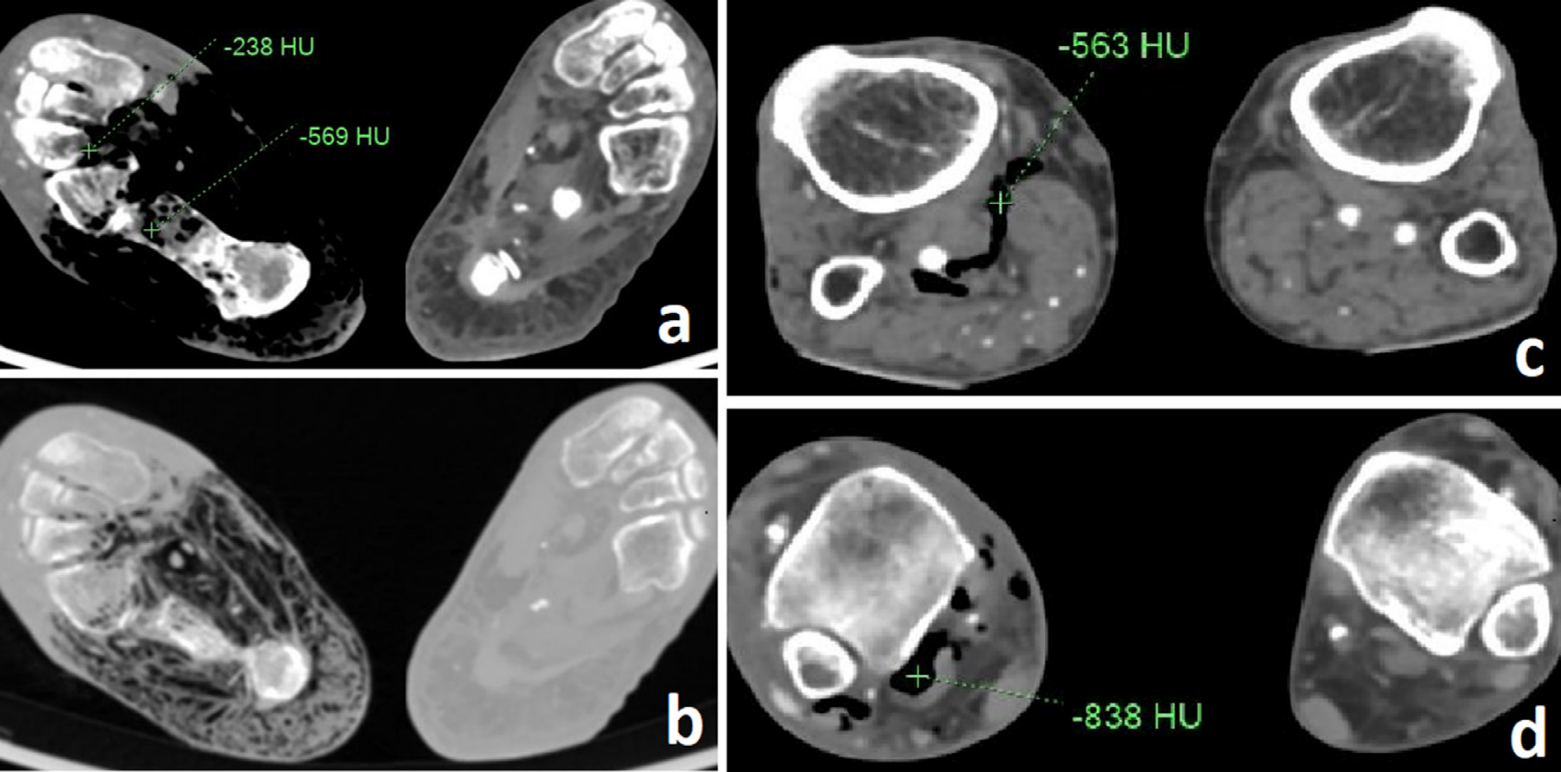
(A) Axial CT images of feet in soft tissue window of a 59-year-old male show cortical erosions and bone destruction in tarsal bones of right foot (calcaneus, cuboid and cuneiforms) with presence of intraosseous gas (indicated by Hounsfield units of air) and gross amount of gas in sub-cutaneous planes of right mid and hind foot. (B) Axial CT image in lung window supports the presence of air as black areas in the involved regions of the right foot. These features are suggestive of “Emphysematous osteomyelitis” involving tarsal bones of right foot with extension to soft tissues. (C and D) Axial CT angiogram images at the level just below knee (C) and at the level just above ankle (D) show presence of gas in deep tissue plane of right leg likely due to tracking up of air/infection from foot; visualized muscles in right leg show more or less normal bulk compared to that on left side. CT angiogram images in C and D also demonstrate normally opacified arteries of the leg without obstruction/thrombosis supporting the infective etiology rather than incited by vascular cause.

**Fig. 2:**
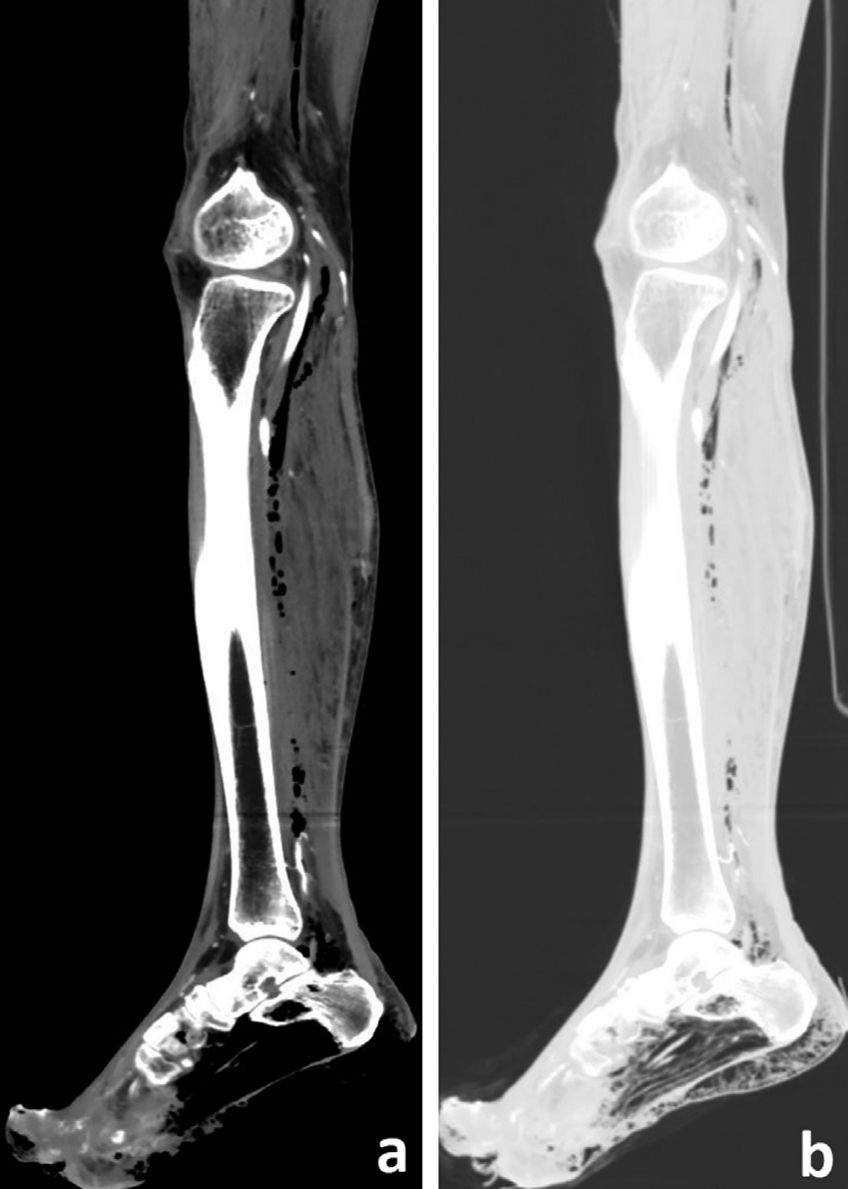
(A) Sagittal CT images of right leg and foot show presence of gas in calcaneus and sub-cutaneous planes of foot with extension of gas to deep intermuscular compartment of left lower leg superiorly up to distal thigh. Intra-osseous gas in calcaneus with extension to soft tissue supports the diagnosis of “Emphysematous osteomyelitis.” (B) Sagittal CT image in lung window supports the findings described above with black areas representing gas.

Ultrasonography/Doppler examination of the bilateral lower limbs did not show significant occlusions in the arteries of bilateral lower limbs; however, >50% stenosis was noted in the right external iliac artery. Computed tomography angiography of bilateral lower legs was then performed on the patient which made it possible to diagnose emphysematous osteomyelitis of right foot. The CT images along with their interpretation have been shown in Figures 1 and 2.

The patient was not taking anti-diabetic medications for about a year and was promptly started on inj. Insulin along with some oral medications for control of blood sugar. The patient was started on broad spectrum intravenous antibiotics and “Posterior flap below knee amputation” surgery was done successfully followed by gradual improvement in the symptoms.

The specimen obtained after the amputation was sent for gram staining and culture, and revealed gram negative bacilli, along with growth of *E coli* on culture. The patient was counseled about the importance of taking medications for his conditions (such as diabetes mellitus and hypertension) and was informed on how uncontrolled levels of glucose and blood pressure were related to the complications he developed.

## Discussion

Emphysematous osteomyelitis is a rare life-threatening condition, characterized by the presence of intraosseous gas [4]. Only a handful of cases have been reported so far, the common locations being pelvis, femur, tibia, fibula, and spine. Foot is the unusual location for the occurrence of emphysematous osteomyelitis [5]. The first case of emphysematous osteomyelitis of foot was described by Mautone et al. in 2014 [2].

The underlying comorbid conditions which compromise the normal immune functions like uncontrolled diabetes mellitus, malignancy, and immunosuppressive therapy are found to be associated with this condition [6]. In our patient, the associated comorbidity was diabetes mellitus for last 20 years.

The most common route of the spread of infection is hematogenous dissemination. However, other rarer modes of disease spread like extension of intraabdominal infection, intraabdominal or spinal surgery, or from skin or soft tissue infection have also been reported [2,5]. The causative organism of the infection can be both aerobic and anaerobic bacteria. The most common cause of infection is the mono/poly microbial infection caused by anaerobes or the members of Enterobacteriaceae family [4,6].

The radiological differential diagnoses of this condition (presence of intraosseous gas), which needs to be excluded before making its diagnosis are penetrating wounds, open fractures, post biopsy status, osteonecrosis, and the lymphangiomatosis of bone [7]. Intravertebral gas may be seen in disc degeneration leading to the development of negative pressure in the intervertebral disk [2].

There is a crucial role of the radiologists in diagnosing this rare condition, determining the nature and the extent of involvement, and enabling implementation of prompt treatment plan [2,5]. Presence of intraosseous gas locules on imaging is pathognomonic for emphysematous osteomyelitis [8]. Plain X-ray film can be useful in detecting the air pockets in the bone and the soft tissue. CT scan is usually done for the confirmation of X-ray findings and is considered as an excellent modality in detection of intraosseous gas [2,5,8]. In our case, the diagnosis of emphysematous osteomyelitis of foot was made based on the CT scan findings.

Emphysematous osteomyelitis requires prompt and aggressive treatment plan. Surgical debridement is the cornerstone in the management of this condition [8]. Intravenous antibiotic therapy is useful in controlling the spread of infection [8]. In our case, the patient was started on broad spectrum intravenous antibiotics and posterior flap below knee amputation was done, which proved to be successful in controlling the spread of infection and prevention of further tissue loss.

## Conclusion

Emphysematous osteomyelitis is a potentially life-threatening condition associated with high mortality and significant morbidity. Early diagnosis and prompt aggressive treatment plan is necessary in order to prevent the potentially devastating consequences. So, the radiologists ought to be aware of identifying intraosseous gas as an alarming sign of emphysematous osteomyelitis.

## Ethical approval

Case reports are exempt from ethical approval in our institution.

## Author contribution

All the authors individually did the final proof-reading of the manuscript before submission.

## Patient consent

Written informed consent was obtained from the patient for publication of this case report and accompanying images. A copy of the written consent is available for review by the Editor-in-Chief of this journal on request.
